# Discovery of the Circulation of the Blood

**Published:** 1859-05

**Authors:** 


DISCOVERY OF THE CIRCULATION OF THE BLOOD.—We understand that a translation, from the pen of our esteemed collaborator, Dr. J. O. Reeve, of Ohio, of Flouren's "History of the Discovery of the Circulation of the Blood," will appear about the first of May, from the publishing house of Rickey, Mallory & Co., of Cincinnati. From the ability of Dr. Reeve, we are confident that the American edition will be a faithful and elegant translation of the French.

